# Inducible Genetic Lineage Tracing of Cortical Hem Derived Cajal-Retzius Cells Reveals Novel Properties

**DOI:** 10.1371/journal.pone.0028653

**Published:** 2011-12-13

**Authors:** Xiaochun Gu, Bin Liu, Xiaojing Wu, Yan Yan, Ying Zhang, Yiquan Wei, Samuel J. Pleasure, Chunjie Zhao

**Affiliations:** 1 Key Laboratory of Developmental Genes and Human Diseases, MOE, Institute of Life Science, Southeast University, Nanjing, Jiangsu, People's Republic of China; 2 Department of Neurology, Programs in Neuroscience, Developmental Biology and Biomedical Sciences, San Francisco, California, United States of America; University of Frankfurt - University Hospital Frankfurt, Germany

## Abstract

During cortical development, Cajal-Retzius (CR) cells are among the earliest-born subclasses of neurons. These enigmatic neurons play an important role in cortical development through their expression of the extracellular protein, reelin. CR cells arise from discrete sources within the telencephalon, including the pallial-subpallial border and the medial (cortical hem) regions of the pallium. Combined evidence suggests that CR cells derived from distinct origins may have different distributions and functions. By tracing CR cells derived from the cortical hem using the inducible Cre transgenic mouse tool, Frizzled 10-CreER™, we examined the specific properties of hem-derived CR cells during cortical development. Our results show that the progenitor zone for later production of CR cells from the hem can be specifically marked as early as embryonic day 6.5 (E6.5), a pre-neural period. Moreover, using our Cre line, we found that some hem-derived CR cells migrated out along the fimbrial radial glial scaffold, which was also derived from the cortical hem, and preferentially settled in the hippocampal marginal zone, which indicated specific roles for hem-derived CR cells in hippocampal development.

## Introduction

Cajal-Retzius (CR) cells are among the earliest-born neurons in the developing cerebral cortex. They are found in the marginal zone, the most superficial layer of the cerebral cortex, and play many pivotal roles in cortical development [1], [2].. The sources, functions and properties of CR cells have been the subject of many recent studies. Several distinct sources of CR cells have been identified, including the cortical hem, the pallial-subpallial boundary (PSB) and the septum [3]. More recently, some CR cells have also been proposed to originate from the thalamic eminence [4], [5].

CR cells derived from Dbx1-positive progenitors in the PSB migrate to the dorsolateral and piriform cortex, but CR cells from the septum largely migrate to the rostral-medial and piriform cortices [3]. The ablation of Dbx1 progenitors with diphtheria toxin fragment A(DTA) starting at embryonic day 11 (E11.0) results in the loss of CR cells in the rostral-medial and dorsolateral pallium, suggesting that CR cells from the PSB have specific functions in early regionalization of the cerebral cortical neuroepithelium [3].

The cortical hem [6] is a major source of CR neurons [3], [7], [8], [9], [10], [11]. Previous studies in hem-ablated Wnt 3a-DTA/Emx1-Cre mice showed surprisingly normal cortical layering. However, the entire hippocampus is missing in these animals, and the mice die shortly after birth, which makes it impossible to study the role of hem-derived CR cells during later cortical developmental stages [9]. Therefore, many of the properties and functions of cortical hem-derived CR cells remain to be further elucidated.

BrdU birth-dating studies in mice have shown that approximately 53% of the CR cells covering the entire cortex are generated between embryonic day (E)10.5 and E11.5 [8]. Interestingly, More than 95% of CR cells in the neocortex die after the first postnatal week. However, a much higher percentage survive in the hippocampus, which suggests additional important roles for CR cells in the postnatal hippocampus [12], [13].

Here, we examined the properties of CR cells from the cortical hem using the inducible Cre transgenic mouse tool, Frizzled 10-CreER™ [11]. In these mice, CR cells are specifically labeled by crossing with ROSA26 reporter mice, and the expression of reporter gene is temporally controlled by tamoxifen (TM) administration. Using these mice, we found that many CR cells originating from the cortical hem preferentially settled in hippocampal marginal zone and migrated along the fimbrial radial glial scaffold, which is also derived from the hem. Remarkably, we also found that the progenitor zone for later CR cell generation from the hem is specified as early as E6.5.

## Results

### Cortical hem-derived CR cells arose from progenitors that expressed Frizzled10

**Figure 1:**
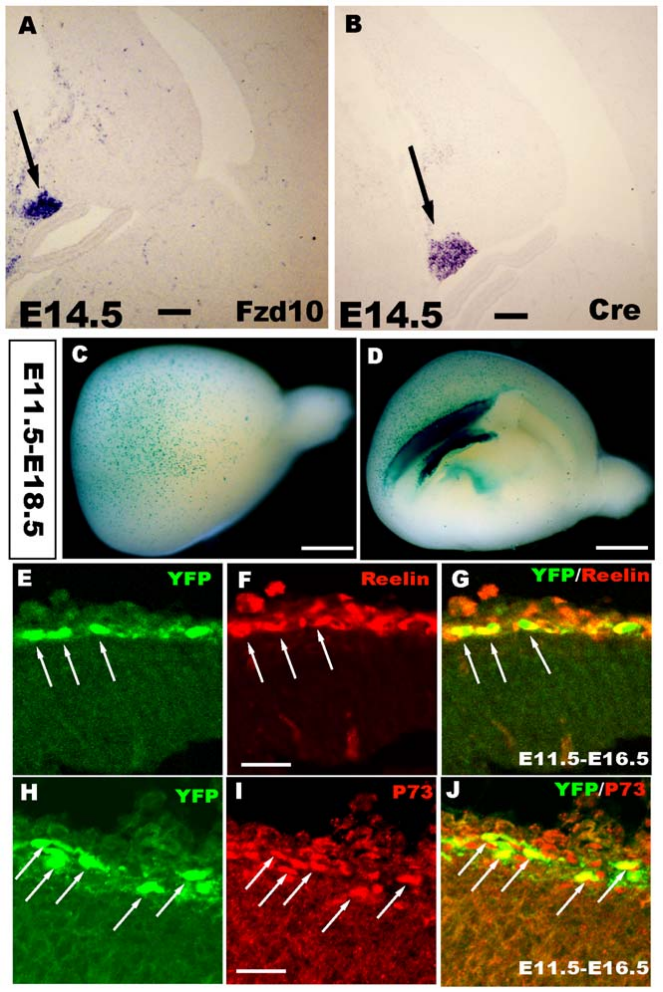
Cortical hem-derived CR cells originating from progenitors expressing Fzd10. A–B: RNA in situ hybridization showing that Cre expression mimics endogenous Fzd10 in the cortical hem in the Fzd10-CreER™ mouse. C–D: Whole-mount X-gal staining of E18.5 Fzd10 CreER™/R26R-LacZ brains when TM was administered at E11.5, showing the output of hem-derived CR cells. C: Lateral view of the hemisphere. D: Middle view. E–J: YFP reporter-positive cells are both reelin^+^ and P73^+^, indicating that these cells are CR cells originating from the cortical hem. E–G: When TM was injected at E11.5 and double immunostaining was performed at E16.5, YFP^+^ cells expressed reelin. H–J: YFP^+^ cells were also P73 positive. E11.5–E18.5, TM was administered at E11.5 and brains were stained at E18.5. Scale bars: A, B: 100 µm; C–D: 2 mm; others: 200 µm.

We previously reported a transgenic mouse line, Frizzled 10-CreER™, in which a fusion protein composed of Cre and a mutated form of the ligand-binding domain of the estrogen receptor, Cre-ER™, were used [11]. In these mice, Cre recombinase is expressed specifically in the cortical hem in the telencephalon and mimics endogenous Frizzled10 (Fzd10) expression (Fig. 1A–B). Because Cre is confined to the Fzd10-expressing hem both spatially and temporally, cells labeled with β-gal or yellow fluorescent protein(YFP) are interpreted as being derivatives of the Fzd10-positive cells. In the present study, the Frizzled 10-CreER™ line was crossed with R26R-LacZ or R26R-YFP reporter mice, and Cre-mediated recombination was induced by administration of TM at specific developmental points. When TM was injected at E11.5 and X-gal staining for the labeling of hem-derived cells was performed at E18.5, a large population of β-gal^−^expressing cells covered the surface of the cortex (Fig. 1C–D). Furthermore, when cells were labeled by the YFP reporter, they were distinguished by the expression of reelin and P73, which were detected by double immunostaining (Fig. 1E–J) and indicated that the hem-derived cells were CR cells from the progenitors that expressed Fzd10 in the cortical hem.

### The progenitor zone for later CR cell generation was specified as early as E6.5, a pre-neural period

**Figure 2:**
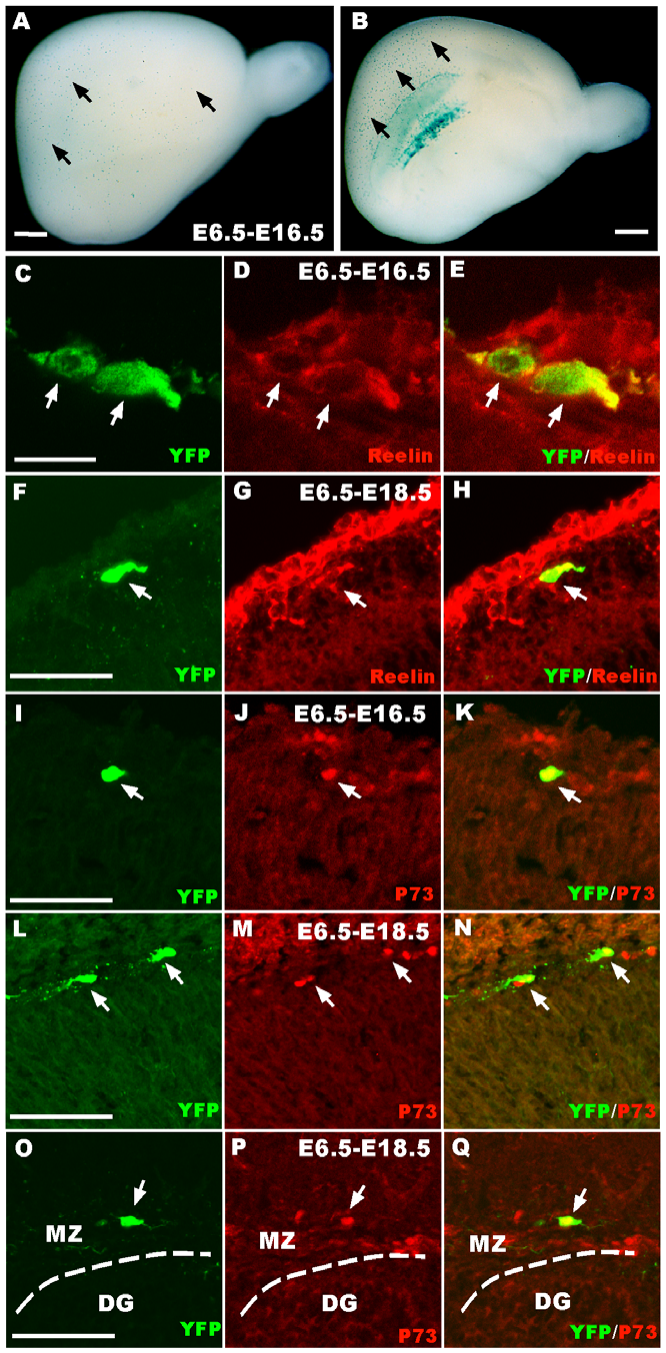
The progenitor zone that will later generate CR cells is specified as early as E6.5. A–B: Whole-mount X-gal staining of E18.5 Fzd10 CreER™/R26R-LacZ brains when TM induction was given at E6.5. Arrows show CR cells migrating out of the cortical hem. C–H: Double immunostaining shows that induced YFP-positive cells are reelin positive at E16.5 (C–E, arrows) and E18.5 (F–H, arrows) in the neocortical MZ. I–N: Double immunostaining shows that induced YFP-positive cells are also P73 positive. O–Q: YFP and P73 are co-localized at the marginal zone of the hippocampal dentate gyrus (arrows). DG, dentate gyrus; MZ, marginal zone. Scale bars:A, B: 1 mm; C–E: 225 µm; others: 200 µm.

**Figure 3:**
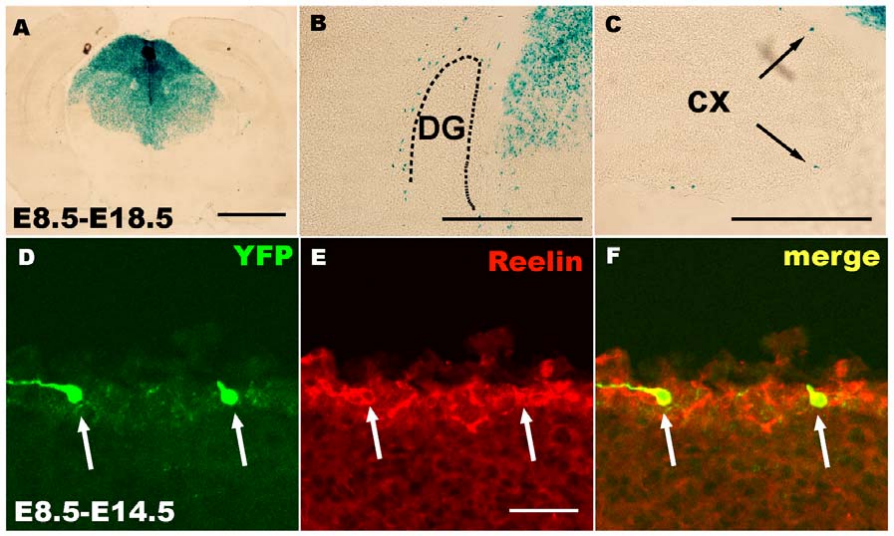
YFP^+^ cells induced by TM administration at E8.5 are reelin-positive CR cells. A–C: X-gal staining on E18.5 Fzd10 CreER™/R26R-LacZ brain sections. B shows β-gal^+^ CR cells settled at the marginal zone of the dentate gyrus. C: Arrows showing the β-gal^+^ CR cells at the marginal zone of the cortex. D–F: Double immunostaining shows that induced YFP-positive cells are reelin-positive at E14.5 when TM was administered at E8.5. DG, dentate gyrus; Cx, cortex. Scale bars: A, 2 mm; B–C: 200 µm; others: 300 µm.

We next examined how early TM administration could result in labeling CR cells and the progenitor zone for CR cells are specified using our transgenic too. To our great surprise, when TM was injected at E6.5, β-gal^+^ cells were observed in the cortical and hippocampal marginal zone at E18.5 (Fig. 2A–B). This result was consistent with that of a previous study that showed Frizzled10 gene expression began at a very early stage [14]. To confirm that the cells were CR cells, we performed yellow fluorescent protein (YFP)/reelin and YFP/P73 double immunostaining at E16.5 and E18.5, respectively. YFP-positive cells were both reelin- and P73-positive, which demonstrated that these YFP-positive cells were CR cells (Fig. 2C–Q). These results indicated that the progenitor zone, which later generates CR cells, was identifiable as early as E6.5. In addition, these results indicated that Fzd10 marked the future cortical hem from very early in forebrain development, probably indicating expression in the lateral neural folds, which will become the roof plate upon neural tube closure. When induced with TM at E8.5, β-gal^+^ cells were also observed to be located in the hippocampal marginal zone and the neocortex, and these cells were also reelin-positive CR cells (Fig. 3A–F). All these results suggest that the progenitor zone that will later generate CR cells is specified at a very early stage of development.

### Hem-derived CR cells preferentially settle in the hippocampal MZ

**Figure 4:**
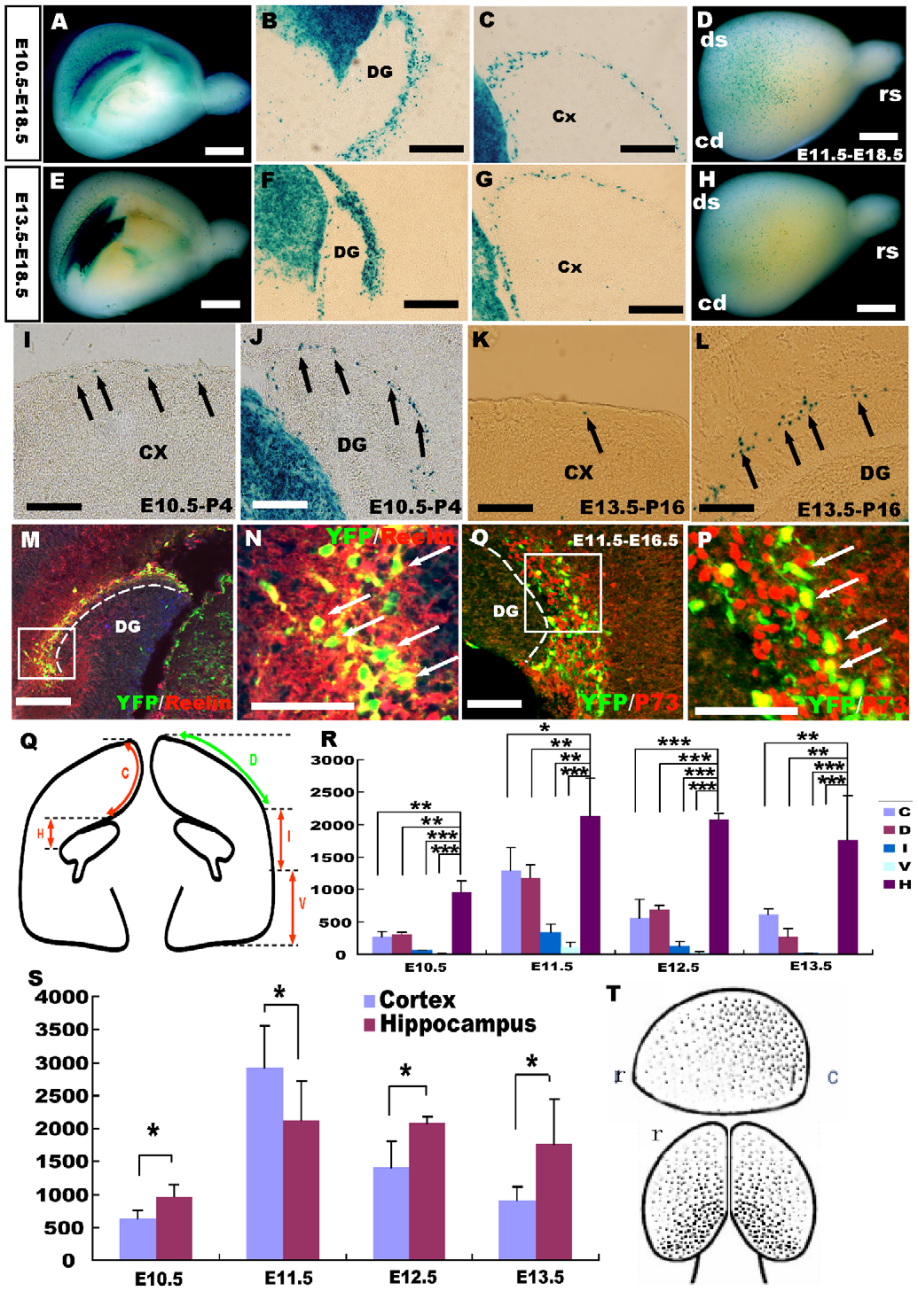
Hem-derived CR cells preferentially settled at the hippocampal marginal zone. A–H: X-gal staining of E18.5 hemispheres when TM injection was given at E10.5 (A–C), E11.5 (D) and E13.5 (E–H). A, E, D, H: The hem-derived CR cells preferentially populated the dorsal-caudal cortical surface. I-L:β-gal^+^ cells can be detected at P4 (I, J) and P16 (K, L) at the hippocampal and neocortical marginal zone. I, K: X-gal staining showing β-gal^+^ cells in P4 and P16 neocortical marginal zones, respectively. J, L: X-gal staining showing β-gal^+^ cells in P4 and P16 hippocampal marginal zones, respectively. M–P: Double staining shows that the YFP-positive cells that settled at the hippocampal marginal zone are both reelin (M, N) and P73 positive (O, P), indicating that these cells are CR cells. Q: Schematic drawing of the cortical and hippocampal areas for CR cell counting, the surface of the cortex was divided into sub-regions of H (hippocampus), D (dorsal cortex),V (ventral cortex),I (intermediate cortex) and C (cingulate cortex) sub-regions. R: Statistical analysis of the distribution of CR cells in sub-regions. S. More β-gal^+^ CR cells populated the hippocampal MZ than other cortical regions (*p<0.05, **P<0.01, ***P<0.001). S Statistical analysis of the total number of CR cells settled at hippocampal and cortical MZ at different developmental stages of E10.5, E11.5, E12.5 and E13.5 respectively (*P<0.05) CR cells preferentially migrated to the hippocampal MZ at most developmental stages. At the stage of E11.5, when the production peak of CR cells appears, the total number of CR cells in the whole cortical MZ seems larger than that of in the hippocampal MZ. However the number of CR cells in the hippocampal MZ (H) is more than any other sub-regions (C, D, I, V) in the cortex as shown in R. T: schematic drawing of the distribution of CR cells derived from the cortical hem. Black dots represent CR cells. H, hippocampus; C, cingulate cortex; D, dorsal cortex; I, intermediate cortex; V, ventral cortex. DG, Dentate gyrus; CX, Cortex. rs, rostral cortex; cd, caudal cortex; ds, dorsal cortex; MZ, marginal zone; r, rostral telencephalon ; c, caudal telencephalon,. Scale bars: A, E, D, H: 2 mm; others: 400 µm.

During cortical development, the production of CR cells at several sites may guarantee rapid coverage of all of the areas of the cerebral cortex by these neurons, thereby ensuring that these cells are in the correct position to accomplish their distinct functions during development. To examine the distribution of hem-derived CR cells, X-gal staining was performed on Frizzled10-CreER™; ROSA26-LacZ brains. The hem-derived CR cells mostly settled in the MZ of the dorsal caudal-medial cortex, preferentially in the hippocampal formation (Fig. 4A–H). When TM induction was performed from E10.5 to E13.5, time points when the largest populations of CR cells are produced, more CR cells populated the hippocampal MZ than other subregions of the cortex (Fig. 4B, C, F, G). Additionally, the hem-derived CR cells can still be detected in the hippocampal formation until at least P16 although the number of cells decreased from P4 to P16 (Fig. 4I–L). Quantitative analysis indicates that approximately 57.1±10.2% of β-gal^+^ CR cells distributed to the hippocampal MZ and 42.9±10.2% of β-gal^+^ CR cells were found on the surface of the cortex. Furthermore, more β-gal^+^ CR cells were located at the hippocampal formation than any other areas of the cortex (Fig. 4R, S). These CR cells that settled in the hippocampal MZ expressed both reelin and P73 (Fig. 4M–P), consistent with previous studies of hem-derived CR cells expressing P73 [15].

### The hem-derived CR cells migrate along the fimbrial radial glial scaffold

**Figure 5:**
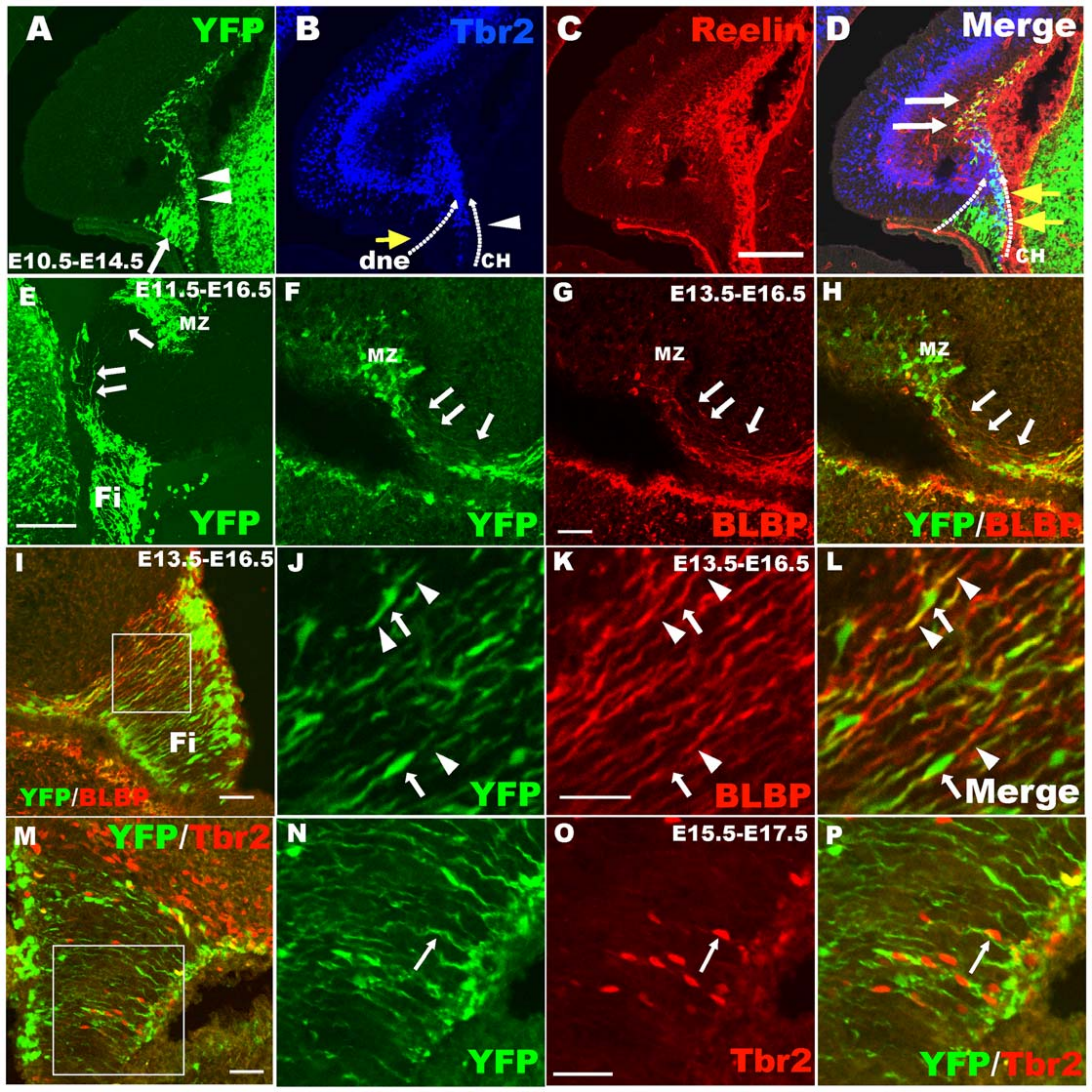
The hem-derived CR cells migrate to the hippocampal marginal zone along the fimbrial radial glial scaffold. A–D: Triple immunostaining for YFP/reelin/Tbr2 in E14.5 brains when TM was injected at E10.5. A: Cortical hem-derived YFP-positive cells consisting of two types of cells, radial glial scaffold (arrow) and migrating cells (arrowheads). B: Anti-Tbr2 staining shows two Tbr2^+^ migrating streams, one from the dentate neuroepithelium to produce dentate granule cells indicated by yellow arrows, the other from the cortical hem (arrowheads). They meet at the area of the future dentate. C: Hem-derived reelin-positive CR cells. D: Yellow arrows indicate double-labeled YFP^+^/Tbr2^+^ cells migrating out of the cortical hem, and white arrows indicate YFP^+^/reelin^+^ cells settled at the MZ zone of the hippocampus. E: Some scaffold-like YFP-positive cells are derived from the cortical hem and distribute to the dentate gyrus from the hem (arrows). F–L: The YFP^+^ scaffold is positive for the radial glial marker BLBP. F–H: YFP^+^/BLBP^+^ radial glial scaffold extend to the DG from the fimbria (arrows). I–L: YFP^+^/BLBP^+^ radial glial scaffold in the fimbria. Arrowheads indicate the processes of the radial glia, and the arrow shows its cell body. J–L show high-magnification views of the area boxed in I. M–P: CR cell progenitors, Tbr2^+^ cells migrate out of the hem along the YFG-positive radial glial scaffold also derived from the cortical hem. Arrows in N–P show migrating Tbr2^+^ CR progenitors. N–P: High-magnification view of the boxed area in M. dnp, dentate neuroepithelium; CH, cortical hem; Fi, fimbria; MZ, marginal zone; DG, dentate gyrus. Scale bars: A–E: 200 µm; others: 50 µm.

CR cells are critical for cortical neuron migration, but very little is known about their own migration. Using our transgenic mouse line, we found that the CR cells originating from the cortical hem tangentially migrated to the cortical and hippocampal MZ in an overall posterior-anterior direction (Fig. 4D, H), which differed from the CR cells derived from the PSB [3]. These migrating neurons preferentially distributed in the hippocampal MZ. In order to explore the route of migration for hem-derived CR cell, Frizzled 10-CreER™; ROSA26 YFP brains were examined by immunostaing for YFP, Tbr2 (an intermediate neuronal progenitor marker), BLBP (radial glia marker) and reelin after TM administration at different developing stages. When immunostained for Tbr2, two neuronal migration streams were observed, including one from the dentate neurepithelium to produce dentate granule cells, and the other from the cortical hem. These migration streams met at the area of the future dentate gyrus (Fig. 5B). Triple staining for YFP/Tbr2/reelin showed when cells migrated out of the hem, YFP^+^ cells transiently expressed Tbr2 and thereafter became Reelin^+^ CR cells after arriving at the hippocampal MZ (Fig. 5A–D). Therefore the population of YFP^+^/Tbr2^+^ cells can be considered as the progenitors for CR cells from the cortical hem migrating to the dentate gyrus (Fig. 5D). Thus, in Frizzled10 CreER™/ROSA-YFP transgenic mouse, the YFP^+^ scaffold originates from the cortical hem and extends to the dentate area (Fig. 5E), and this YFP^+^ scaffold is BLBP positive, a marker for radial glial (Fig. 5F–L). Tbr2^+^ CR cell progenitors migrated along the YFP^+^ scaffold can be detected (Fig. 5M–P). These data suggest hem-derived CR cells migrate to the hippocampal MZ along this fimbrial radial glial scaffold.

### The fimbrial radial glial scaffold is necessary for CR cells migration

**Figure 6:**
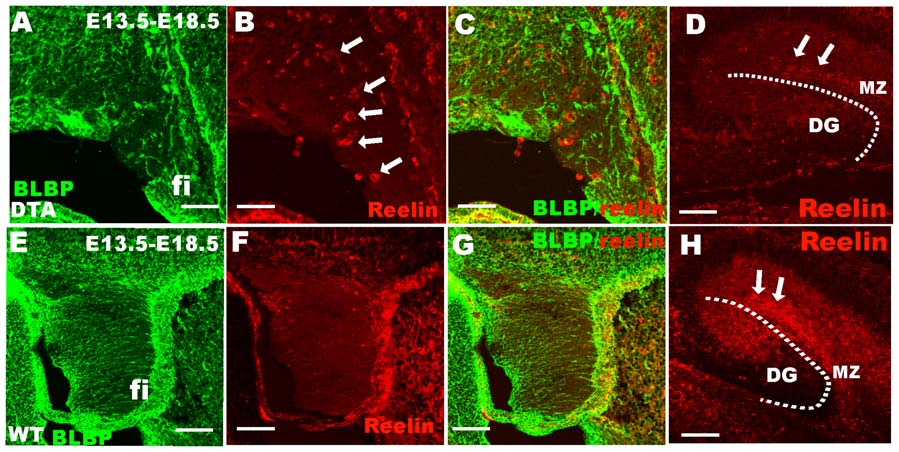
The fimbrial radial glial scaffold derived from cortical hem is necessary for CR cell migration. Reelin-positive CR cells cannot migrate out and ectopically settled at the fimbrial area after the radial glial scaffold is disrupted by DTA toxin expression. A shows that the BLBP-positive radial glial scaffold is disrupted in Fzd10 CreER™/ROSA26DTA mice compared to control mice in E. B shows that reelin-positive CR cells (arrows) were arrested at the fimbria after the disruption of the fimbrial radial glial scaffold by DTA expression, but reelin-positive cells could not be detected in the control fimbria in F. D The number of Reelin^+^ cells (arrows) in Fzd10 CreER™/ROSA26DTA hippocampal MZ was dramatically decreased compared to control. Fi, fimbria; MZ, marginal zone; DG, dentate gyrus. Scale bars: D, H: 100 µm; others: 50 µm.

In order to further test the role of the fimbrial radial glial scaffold in CR cells migration, Frizzled 10 CreER™ mice were crossed with ROSA-DTA in which functional DTA is expressed exclusively upon Cre-mediated recombination [16]. We found that in Frizzled 10 CreER™/ROSA-DTA mice, the fimbrial radial glial scaffold was severely disrupted compared to wild-type (WT) mice (Fig. 6A, D), and many reelin^+^ CR cells ectopically arrested in the fimbria (Fig. 6B, C, E, F), indicating that the fimbrial radial glial scaffolding was pivotal for the migration of hem-derived CR cells. These data also suggested that cortical extrinsic factors may be not required for the maturation of hem-derived CR cells.

### Hem-derived CR cells survive to postnatal stages

**Figure 7:**
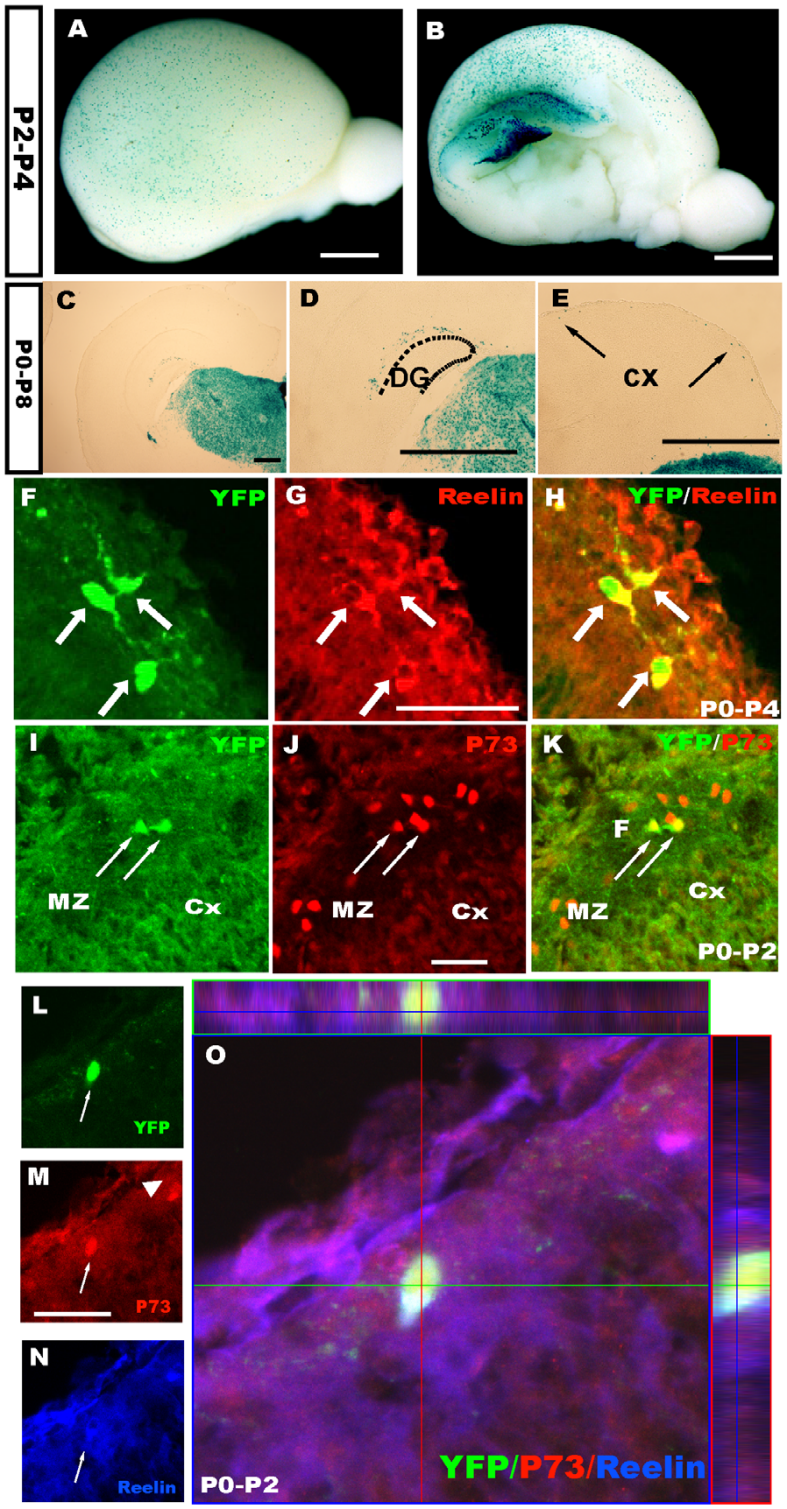
Hem-derived CR cells survive to postnatal stages. A–B: Whole-mount X-gal staining of Fzd10 CreER™/R26R-LacZ brains when TM induction was performed at P2 and examined at P4. C–E: X-gal staining on P8 brain coronal sections showing hem-derived β-gal^+^ cells located at the MZ of the dentate gyrus (D) and neocortex (E) when TM was administered at P0. F–H: YFP^+^ cells induced postnatally were reelin positive. I–K: YFP^+^ cells were also P73 positive, indicating that these cells are CR cells. L–O: Z-stack magnification views show that the YFP-positive cells express the CR cell markers P73 and reelin. P2–P4, TM was administered at P2, and brains were stained at P4, the same as P0–P8. DG, dentate gyrus; Cx, cortex. Scale bars: A–B, 2 mm; others: 200 µm.

To further elucidate the properties of the hem-derived CR cells, the Frizzled 10-CreER™ line was crossed with reporter mice, and TM was administered at different developmental stages. We have found there are still CR cells that can be labeled by β-gal reporters even postnatally (Fig. 7 A–E). Most of these “postnatally labeled” cells are both reelin and P73 positive and located in the cortical marginal zone (Fig. 7F–O). In addition, approximately 87±3% of postnatally induced YFP^+^ cells were P73-positive when TM was injected at P0 and examined at P2, which indicated that these cells were CR cells (Fig. 7F–O). To test whether these CR cells were new born cells being generated by cell division at postnatal stages, BrdU birth-dating was performed. A single dose of BrdU was administered following TM induction at P0, and double immunostaining for anti-BrdU and GFP was carried out at P4. However, few GFP^+^ cells were found to be co-labeled by BrdU (data not shown), suggesting that these CR cells were not newborn cells, but their presence may have been due to residual small amounts of Cre in postmitotic cells. However, these data indicate that some CR cells from the cortical hem can survive to postnatal stages.

## Discussion

The properties of CR cells have been controversial in part due to inconsistent criteria for their morphology and gene expression. CR cells were defined as reelin-immunoreactive cells in the marginal zone in a previous study [7]. Later, P73 was used as a novel marker for CR cells derived from the cortical hem in the present study [15]. These definitions reduce confusion but reflect the complexity of early corticogenesis and postnatal development.

*Tbr1*, *Pax6*, *Emx1/2* and *Lhx5* are important in the controlling CR cell differentiation, migration and survival [17], [18], [19], [20]; however, the molecular mechanism underlying cortical hem-derived CR cell development has not been elucidated. In the developing cerebral cortex, the cortical hem is a major source of CR cells, and is rich in bone morphogenetic proteins (BMPs) and the wingless-Int gene (Wnt) family [6] but is lacking in forkhead box G1 (Foxg1) [21]. Previous work has shown that Foxg1 plays a key role in fate determination of CR cells. Cortical progenitors fail to generate later-born neurons and instead continue to produce CR cells in the absence of Foxg1. Moreover, after the conditional inactivation of Foxg1, deep-layer progenitors revert to CR cells [22]. A cell ablation study with a *Wnt3a*-driven DTA mouse line resulted in the loss of most of the CR cells in the developing cortex [9]. By tracing hem-derived CR cells using the Frizzled 10-CreER™ transgenic line, we demonstrated that hem-derived CR cells arose from Frizzled10-positive progenitors, suggesting that the Wnt signaling pathway may be important for CR cell development. More interestingly, our results indicate the progenitor zone for later CR cells production is specified from as early as E6.5. It has been reported that Foxg1 inhibits the Wnt pathway [23], while Foxg1 is not expressed until E9.5. These data suggest that if Wnt signaling is involved in specifying CR cells, it may occur earlier than Foxg1 is expressed. Foxg1 alone may not be sufficient for the specification of CR cell fate. Recent gene expression profiling of CR cells has identified a number of genes that are expressed in these pioneer neurons [24]. These studies, together with our results may provide the clues to identify the molecular pathways that control the hem-derived CR cell development.

In the developing cerebral cortex, neurons are born on a predictable schedule, and the timing mechanism is programmed with individual progenitor cells. A previous study has suggested that the subplate and cortical plate cells come from a common progenitor [25]. However a common ancestry for preplate cells and CR cells has not been reported, they may be pre-fated separately during a very early developmental time point [25]. Our fate mapping study indicates that CR cells may be pre-fated separately from the preplate cells as early as E6.5. Further understanding these extremely early events will be quite important for cortex development.

In the present study, we have found the hem-derived CR cells migrate along the fimbrial radial glial scaffold, and preferentially settled at the hippocampal marginal zone. In addition, disruption of the fimbrial radial glial scaffold causes the arrest of CR cells in the fimbria. Ablation of the hem does not alter neocortical layering but leads to the deletion of the hippocampus [9]. However, because the mice died shortly after birth, the role of the hem-derived CR cells in hippocampal development needs to be further elucidated. Our results showed that hem-derived CR cells preferentially settled in the hippocampal MZ, indicating that they may be pivotal for the development of the hippocampus. In addition, although the number of hem-derived CR cells in the neocortex decreased rapidly after birth, many remained alive in the hippocampal MZ, suggesting that these hem-derived CR cells may play more important roles in postnatal hippocampal development.

CR cells and their secreted protein, reelin, are important for cell positioning, path-finding and branching of entorhinal afferents during hippocampus formation [13], [26], [27]. Previous studies have indicated that the cortical hem is required for hippocampal induction and expansion, and ectopic hem cells can induce ectopic hippocampal tissue, thus providing evidence that the hem is a hippocampal organizer [21]. However, understanding how the cortical hem organizes hippocampal development has been difficult. Our results showing that hem-derived CR cells preferentially settle at the marginal zone of the developing dentate gyrus, indicating that hem-derived CR cells may play more important roles in dentate gyrus development.

Previous studies have demonstrated CR cells migrate continuously to the MZ of the hippocampus and neocortex after their birth, and more than 80% of CR cell are born during the time window of E10.5–13.5, the production peak of CR cells appears at E11.5, however the portion of CR cells born later than E13.5 are very small [8], [28]. The reason that we chose this specific developmental stage of E13.5 to give tamoxifen is because most CR cells have born at this time and large amount of fimbrilar radial glial scaffold begin to extend from the hem as showing in Fig. 5. Thus the ablation by DT-A expression will mainly disrupt the fimbrilar radial glial scaffold, CR cells born earlier than E13.5 will remain untouched, only a very small portion of. CR cells might be ablated.

The hippocampus is crucial for higher brain functions such as learning and memory. In addition, reelin deficiency in human beings leads to lissencephaly, malformations of the hippocampus and cerebellum and severe epilepsy [29]. Furthermore, decreased level of reelin expression in the hippocampus is related to schizophrenia. These findings suggest the possible involvement of hem-derived CR cells and its secreted protein reelin in the pathogenesis of certain mental disorders [30]. Understanding the development and roles of hem-derived CR cells may help to shed lights on the mechanisms underlying these disorders.

## Materials and Methods

### Animals

The Frizzled 10-CreER™ transgenic mouse line was generated as described previously [11]. ROSA26-LacZ, ROSA26-YFP and ROSA26-DTA mice were purchased from the Jackson laboratory. Timed-pregnant females were obtained by placing male and female mice together overnight. The day of vaginal plug detection was defined as E0.5, and the day of birth was defined as postnatal day (P) 0. All animals were bred in the animal facility at Southeast University. All studies were performed according to protocols approved by Southeast University.

### X-Gal Staining

Mice were perfused transcardially with 2% paraformaldehyde (PFA) in 0.1 M phosphate buffer (PBS), pH 7.4. The brains were removed from the skulls, post-fixed for 30 min at 4°C, cryoprotected in 30% sucrose and sectioned on a Leica CM3050S. After permeabilizing the sections with PBS containing 0.02% NP-40, 2 mM MgCl_2_ for 30 min, the sections were stained with 1 mg/ml X-gal at 37°C for approximately 6–16 h, re-fixed in 4% PFA and coverslipped.

### Immunohistochemistry

Embryos were fixed in 4% PFA, cryoprotected in 30% sucrose and sectioned on a Leica CM3050S. Sections were then washed in PBS, blocked by 10% normal goat serum, permeabilized in PBS containing 0.1% Triton X-100 (PBT) for 2 h and incubated in primary antibody diluted in blocking solution overnight at 4°C. Sections were subsequently washed in PBT, incubated in the secondary antibody for 2 h at 37°C, and washed with PBS five times. As a last step, coverslips were applied. The following antibodies and reagents were used for immunostaining: mouse anti-reelin (Chemicon, MAB536, 1∶1000), mouse anti-calretinin (Chemicon, MAB536, 1∶2000), rabbit anti-BLBP (Abcam, AB32423, 1∶250 ), rabbit anti-P73 (Santa Cruz, sc-7957, 1∶50 ), rabbit anti-Tbr2 (Abcam, AB23345, 1∶800 ), rabbit anti-Cre (Covance, PRB-106C, 1∶2000), FITC goat anti-Rabbit IgG (Jackson Immuno-Research, 111-095-144, 1∶200), FITC goat anti-mouse IgG (Jackson ImmunoResearch, 115-095-146, 1∶200) and goat anti-rabbit Alexa Fluor 633 (Molecular Probes, A21071, 1∶200).

### In situ hybridization

Brains were fixed in 4% paraformaldehyde overnight, cryoprotected in 30% sucrose /DEPC-PBS at 4°C, and then embedded in OCT. Coronal sections were obtained with a Leica CM 3050S cryostat and stored at 70°C until use. In situ hybridization on sections was performed as described previously [10]. The concentration of the probe was 1 µg/µL.

### Tamoxifen Injection

TM (Sigma, T5648) was dissolved in corn oil (Sigma, C8267) at a concentration of 10 mg/ml, and 2 mg TM/40 g body weight was injected into pregnant mice at the developmental stages of E10.5, 13.5 and 16.5 at E6.5, 1 mg TM/40 g body weight was injected.

### Microscopic analysis

Sections were examined with an Olympus (Tokyo, Japan) BX61 microscope equipped with appropriate filter sets and a digital camera (DP71) or confocal microscope (FV1000; Olympus). Stacks of whole-mount brain staining images were captured using a stereoscopic fluorescence microscope (MZFL III, Leica). The stacks were combined into one resulting picture that was reconstructed in a three-dimensional illustration.

### Quantification

To quantify the distribution of hem-derived CR cells, X-gal staining was performed on E18.5 Frizzled10-CreER™; ROSA26-LacZ brains, when TM was injected at E10.5, E11.5 E13.5 respectively. A minimum of 3 embryos per stage were examined. β-gal^+^ positive cells on sixteen sections of each brain were countered from the rostral to caudal hippocampal formation. Images were obtained from an Olympus DP71 microscope. Values are shown as mean ± SEM (number of cells). Statistical analysis was performed using two-tailed *t* test.
